# Odor identification score as an alternative method for early identification of amyloidogenesis in Alzheimer’s disease

**DOI:** 10.1038/s41598-024-54322-3

**Published:** 2024-02-26

**Authors:** Yukifusa Igeta, Isao Hemmi, Kohei Yuyama, Yasuyoshi Ouchi

**Affiliations:** 1 Department of Dementia, Dementia Center, Federation of National Public Service Personnel Mutual Aid Associations, Toranomon Hospital, 2-2-2 Toranomon, Minato-ku, Tokyo, 105-8470 Japan; 2 Division of Dementia Research, Okinaka Memorial Institute for Medical Research, 2-2-2 Toranomon, Minato-ku, Tokyo, 105-8470 Japan; 3 Japanese Red Cross College of Nursing, 4-1-3 Hiroo, Shibuya-ku, Tokyo, 150-0012 Japan; 4 Lipid Biofunction Section, Faculty of Advanced Life Science, Hokkaido University, Kita-21, Nishi-11, Kita-ku, Sapporo, 001-0021 Japan

**Keywords:** Neuroscience, Neurology, Diagnostic markers

## Abstract

A simple screening test to identify the early stages of Alzheimer’s disease (AD) is urgently needed. We investigated whether odor identification impairment can be used to differentiate between stages of the A/T/N classification (amyloid,  tau, neurodegeneration) in individuals with amnestic mild cognitive impairment or AD and in healthy controls. We collected data from 132 Japanese participants visiting the Toranomon Hospital dementia outpatient clinic. The odor identification scores correlated significantly with major neuropsychological scores, regardless of apolipoprotein E4 status, and with effective cerebrospinal fluid (CSF) biomarkers [amyloid β 42 (Aβ42) and the Aβ42/40 and phosphorylated Tau (p-Tau)/Aβ42 ratios] but not with ineffective biomarkers [Aβ40 and the p-Tau/total Tau ratio]. A weak positive correlation was observed between the corrected odor identification score (adjusted for age, sex, ApoE4 and MMSE), CSF Aβ42, and the Aβ42/40 ratio. The odor identification score demonstrated excellent discriminative power for the amyloidogenesis stage , according to the A/T/N classification, but was unsuitable for differentiating between the p-Tau accumulation and the neurodegeneration stages. After twelve odor species were analyzed, a version of the score comprising only four odors—India ink, wood, curry, and sweaty socks—proved highly effective in identifying AD amyloidogenesis, showing promise for the screening of preclinical AD.

## Introduction

Alzheimer’s disease (AD) is the most prevalent cause of dementia and a substantial challenge to healthcare, medical, and socioeconomic systems worldwide. AD accounts for 60–80% of dementia cases. Approximately 6.7 million older patients in the United States are affected by AD^1^. Without improvement in available therapies, this number could increase to 13.8 million by 2060^1^. The economic value of unpaid caregiving for patients with dementia in the US was approximately $339.5 billion in 2022^1^.

AD pathology is characterized by accumulation of amyloid β (Aβ) protein in extracellular senile plaques and of intracellular neurofibrillary tangles (NFTs) composed of phosphorylated Tau (p-Tau)^2^. Aβ accumulation begins approximately 25 years before the onset of symptoms, whereas p-Tau accumulation starts approximately 10 years before symptom onset^3^. The sequence of events from Aβ accumulation to subsequent p-Tau-induced neurotoxicity and neuroinflammation by activated microglia is known as the amyloid cascade hypothesis^2,4^ and has been the basis for the development of disease-modifying therapies^5^.

In 2011, the US National Institute on Aging/Alzheimer’s Association (NIA-AA) classified AD into three stages: preclinical stage, mild cognitive impairment (MCI) due to AD, and AD dementia. Preclinical AD can be diagnosed based on Aβ42 levels in the cerebrospinal fluid (CSF)^6^. The following biochemical biomarkers for AD diagnosis have been established: decreased CSF Aβ42 levels, decreased Aβ42/40 ratio, increased p-Tau/Aβ42 ratio, and increased total Tau (t-Tau)/Aβ42 ratio^7,8^. Plasma biomarkers such as the Aβ42/amyloid precursor protein (APP) ratio determined using immunoprecipitation/mass spectrometry^9^ and pTau181 levels quantified using an ultrasensitive automated enzyme-linked immunosorbent assay (ELISA) system^10^ have also been developed. As a result, AD diagnosis has shifted from being based primarily on clinical assessments to relying on biochemical markers.

In 2016, the NIA-AA proposed the classification system known as A/T/N based on Aβ (A), p-Tau (T), and neurodegeneration (N). This system has been widely adopted to categorize patients with AD according to their biomarker profiles^11,12^ and has shown promising results in the prediction of disease progression and the identification of intervention targets^11,13^. However, concerns have been raised about the accuracy of the A/T/N classification due to the variability in the CSF measurements among different facilities^14,15^. Particularly, Aβ42 is easily adsorbed, and its abundance may therefore be underestimated^14^. This variability in measurement may lead to misclassification of some healthy individuals as amyloid positive.

Lumbar puncture for CSF collection and positron emission tomography (PET) are commonly performed to identify the biomarker required for the A/T/N classification^11^. However, these methods have limitations, including invasiveness, high costs, and radiation exposure. Therefore, alternative, less invasive, more cost-effective screening tests are required. One of the most promising is the odor identification score, which is a well-established early marker for AD^16^. However, there are no available reports on the suitability of the odor identification score for categorization based on the A/T/N classification system. In this study, our objective was to assess the effectiveness of the odor identification score for early AD diagnosis.

## Methods

### Participants

This single-center prospective study was conducted in Japan in compliance with the Declaration of Helsinki. The Clinical Research Ethics Committee of Toranomon Hospital approved the study protocol (Clinical Research No. 1388). A total of 150 Japanese participants of both sexes were recruited. All participants provided written informed consent before the study and underwent physical and neurological examinations at the outpatient dementia clinic of the Toranomon Hospital in Tokyo between April 2017 and April 2021. Eighteen potential participants were excluded because they met at least one of the exclusion criteria, which were as follows: having sample artifacts [bloody CSF (one participant)] or additional diseases [dementia with Lewy bodies (DLB) (seven participants), frontotemporal dementia (FTD) (one participant), multiple system atrophy (one participant), depression (two participants), bipolar disorder (two participants), alcoholism (one participant), severe paranasal sinusitis (two participants), normal-pressure hydrocephalus (one participant), vascular dementia, epileptic seizures, hepatic encephalopathy, postoperative sinus, hypothyroidism, or any blood diseases], heavy smoking, or treatment with antiplatelet or anticoagulant drugs.

All participants underwent physical and neurological examinations and neurological testing, including the Mini-Mental State Examination (MMSE), Alzheimer-Disease Assessment Scale-Cognitive-Japanese version (ADAS-cog J), Frontal Assessment Battery (FAB), 15-item Geriatric Depression Scale (GDS), and Wechsler Memory Scale-revised (WMS-R). Brain function was evaluated based on scores from the attention and concentration (A/C), delayed recall (DR), and logical memory II (LM-II) subtests of the WMS-R. Additional imaging studies were performed, including magnetic resonance imaging (MRI) and ^123^I N-isopropyl-p-iodoamphetamine brain perfusion single-photon emission computed tomography (SPECT). Patients with DLB were excluded based on ^123^I-metaiodobenzylguanidine (MIBG) and dopamine transporter (DAT) scans. These tests were conducted in cases exhibiting symptoms such as olfactory dysfunction, constipation, rapid eye movement sleep behavior disorder, hallucinations, extrapyramidal symptoms, altered consciousness, or posterior cerebral hypoperfusion assessed via SPECT. Participants with MIBG H/M ratios below 2.2 or with DAT striatal or putaminal binding ratios of − 2.0 z-scores or lower were classified as having neurodegenerative parkinsonism and excluded from the study^17^.

AD was diagnosed based on the Diagnostic and Statistical Manual of Mental Disorders-Fourth Edition Text Revision (DSM-IV-TR) and the National Institute of Neurological Communicative Disorders and Stroke–Alzheimer’s Disease and Related Disorders Association clinical definition^18^ of probable AD. Participants with AD diagnosed by these diagnostic criteria had MMSE scores ranging from 9 to 30 and ADAS-cog J scores of 7 or higher. In contrast, aMCI was diagnosed according to the clinical criteria of the Alzheimer’s Association Workgroup of the National Institute on Aging and classified as amnesia^19^. Participants with memory complaints but no significant impairment in daily functioning were classified as having aMCI if they scored between 23 and 30 on the MMSE and 12 or lower on the ADAS-cog J and were > 1.0 standard deviations below the age- and education-adjusted cutoff scores on the DR portion of one LM-II of the WMS-R. Healthy controls without cognitive impairments had normal daily functioning, scored between 25 and 30 on the MMSE and 8 or lower on the ADAS-cog J, and were within 1.0 standard deviations of the age- and education-adjusted cutoff scores on the DR portion of one LM-II story of the WMS-R.

All neuropsychological tests and imaging diagnoses mentioned above were used as clinical aids by an experienced neurologist and geriatrician, who strictly adhered to the diagnostic criteria.

### Odor identification test

The Open Essence (OE) test (Fujifilm Wako Pure Chemicals Corporation, Osaka, Japan) was employed to measure odor identification ability. The OE test was specifically developed to assess the odor identification ability in the Japanese population. This card-based odor identification test involves the microencapsulation of the same 12 odors featured in the Smell Stick Identification Test (OSIT-J) in a solid cream form. It comprises a total of 12 odors, including Indian ink (borneol), wood (essential oil), perfume, menthol (menthol), Japanese orange (artificial flavor), curry (natural flavor), cooking gas (tetrahydrothiophene), rose, hinoki (Japanese cypress essential oil), sweaty socks (isovaleric acid), condensed milk (artificial flavor), and roasted garlic (natural flavor). Each card folded in two contains microcapsules containing one of the 12 odors on the inner adhesive surface. The microcapsulated odor is released upon unfolding of the card. The participant was then required to select one of the six printed answers (four different odors, “unknown,” and “odorless”) in the manner of a choice task with six alternatives. Its user-friendly nature makes this test suitable for outpatient clinics, and it has been widely used in recent olfaction studies carried out in Japan^20^.

### Collection of CSF and plasma samples

CSF and plasma samples were obtained from participants. These samples were not collected from patients who had been treated with antiplatelet or anticoagulant drugs or had any blood diseases. The participants were instructed to have their last meal at 9:00 p.m. on the night before the lumbar puncture, refrain from consuming alcohol, and sleep for at least 6 h. Lumbar punctures were performed between 9:00 and 10:00 a.m. A 23-25G top spinal needle with stainless steel and a polypropylene base was used to collect CSF into a sterile 10ml polypropylene spit tube. All procedures were performed by an experienced physician using the same technique and equipment. The collected CSF was centrifuged at 2200 × *g* for 7 min at 20 °C to precipitate the cells and other insoluble materials. The resulting supernatant was separated in 500 µl aliquots into 1.5 ml polypropylene microtubes. Following CSF collection, whole blood was collected from the left upper arm in a vacuum tube containing EDTA 2Na. The plasma was centrifuged at 2200 × *g* for 15 min at 4 °C and stored in 500 µl aliquots. All samples were stored at − 80 °C within 1 h of collection until analysis. Only one freeze–thaw cycle was performed.

### ELISA

The concentrations of Aβ1-40, Aβ1-42, Tau phosphorylated at threonine 181 (p-Tau181), and t-Tau in CSF were determined using commercially available ELISA kits. Specifically, Human β Amyloid (1–40) and Human β Amyloid (1–42) ELISA Kits (Fujifilm Wako, Osaka, Japan) were used to measure Aβ1-40 and Aβ1-42 levels, respectively. TAU (pT181) Human ELISA Kit and TAU (Total) Human ELISA Kit (Thermo Fisher Scientific, Waltham, MA, USA) were used to measure p-Tau181 and t-Tau levels, respectively. These ELISA kits employ enzyme antibody labeling for the colorimetric detection of the target molecules.

### Identification of the apolipoprotein E phenotype

The apolipoprotein E (ApoE) phenotype was identified by separating plasma samples using isoelectric electrophoresis, probing with an ApoE polyclonal antibody, and performing western blot analysis using an ApoE isoelectric focusing system (JOKOH, Tokyo, Japan). For sample loading, a pre-treatment solution was prepared by combining 10 μl of the sample with 100 μl of preparing solution. This pre-treatment solution was then applied to 3.5 × 10 mm filter paper for sample application and allowed to absorb at 22 °C for 15 min. Samples of 3/3 and 2/4 phenotypes were concurrently run in each gel as a routine verification measure (Supplementary Fig. S1).

### Statistical analyses

To investigate the correlations and associations among the odor identification scores, neuropsychological test scores, and CSF biomarkers, we employed Pearson’s correlation to assess linear relations, Spearman’s correlation to assess nonlinear relations, and chi-squared tests to assess associations between categorical variables. One-way analysis of variance (ANOVA) or Kruskal–Wallis tests were performed to compare three or more groups. If multiple comparisons were necessary as a post-hoc analysis, the Tukey Kramer test was used after ANOVA, and the Scheffe test was employed after the Kruskal–Wallis test.

Receiver operating characteristic (ROC) analysis was performed using R (version 4.1.1; R Foundation for Statistical Computing, Vienna, Austria) to determine the diagnostic cutoff values for CSF markers in patients with AD versus patients with aMCI or healthy individuals with normal cognition (Table 1, Supplementary Fig. S2). The ROC curve in this context is presented in Supplementary Fig. S2. The cutoff value was defined as the value corresponding to the point on the ROC curve that minimizes the Euclidean distance to the coordinates (0, 1). Specificity and sensitivity were calculated based on the coordinates of that point. Based on lower Aβ42 (A+), higher p-Tau (T+), and higher t-Tau (N+) than the respective cutoff values, eight A ± /T ± /N ± groups were defined (Table 2, Supplementary Fig. S3).

**Table 1:** **ROC analysis according to clinical classification for each biomarker**.

Biomarker	AUC	95% CI (DeLong)	Cut-off	Specificity	Sensitivity
Aβ42	0.825	0.751–0.900	658.48 pg/ml	0.855	0.714
p-Tau181	0.721	0.632–0.810	58.37 pg/ml	0.602	0.796
t-Tau	0.721	0.62–0.822	756.6 pg/ml	0.771	0.633
Aβ42/Aβ40	0.777	0.692–0.863	0.103	0.723	0.776
p-Tau/Aβ42	0.836	0.769–0.903	0.063	0.675	0.898
Odor identification scores	0.785	0.706– 0.864	4.5	0.795	0.673

ROC analysis to discriminate between normal + aMCI and AD patients. AUC and cutoff values for AD diagnosis for each biomarker were established.

*AD* Alzheimer’s disease; *aMCI* amnestic mild cognitive impairment; *CI* confidence interval; *ROC* receiver operating characteristic; *AUC* area under the ROC curve.

**Table 2:** **A/T/N classification of the study population**.

Variables	Total	Normal	AD continuum				Suspect non-AD pathophysiology (SNAP)			*P*-value
		A−/T−/N−	A+/T−/N−	A+/T+/N−	A+/T+/N+	A+/T−/N+	A−/T+/N−	A−/T+/N+	A−/T−/N+	
Number	132	37	6	13	22	5	27	10	12	–
Number: normal, aMCI, AD	60,23,49	30,6,1	2,0,4	0,5,8	1,3,18	0,1,4	15,6,6	3,0,7	9,2,1	–
Age (years)	71.2 (7.5)	70.3 (7.5)	68.0 (8.9)	72.1 (6.0)	70.7 (8.2)	70.4 (10.8)	71.4 (8.2)	75.9 (3.9)	71.0 (5.9)	0.389**
Male sex (%)	67/132 (51)	20 (54)	2/6 (33)	4/13 (30)	11/22 (50)	1/5 (20)	16/27 (59)	5/10 (50)	8/12 (67)	–
Education, years	14.4 (2.2)	14.4 (1.9)	14.3 (2.0)	14.8 (2.9)	13.8 (2.0)	13.0 (3.0)	14.8 (2.0)	13.7 (2.4)	15.2 (2.6)	0.390**
ApoE4 carrier number (%)	44/131 (34)	3/37 (8)	2/6 (33)	8/13 (62)	14/22 (64)	2/5 (40)	7/26 (27)	5/10 (50)	3/12 (25)	–
BMI	22.2 (3.7)	22.9 (3.6)	24.7 (5.7)	20.2 (3.0)	20.6 (3.4)	20.8 (4.9)	23.7 (3.6)	21.6 (3.2)	22.1 (2.5)	0.012
MMSE	26.5 (3.9)	28.9 (1.3)	22.7 (4.9)	25.2 (2.9)	24.6 (3.8)	22.6 (3.8)	27.2 (3.1)	22.6 (6.0)	28.8 (1.4)	< 0.001**
ADAS-cog J	7.3 (5.8) (n = 128)^a^	3.8 (2.1) (n = 37)	14.9 (9.8) (n = 6)	9.9 (4.4) (n = 13)	10.3 (4.6) (n = 21)	17.4 (13.4) (n = 5)	5.7 (3.4) (n = 25)	8.9 (3.9) (n = 9)	3.9 (2.1) (n = 12)	< 0.001**
FAB	15.1 (2.8)	16.5 (1.8)	12.5 (3.2)	13.4 (2.9)	14.0 (3.3)	13.4 (2.6)	15.4 (2.4)	14.3 (2.9)	16.7 (1.8)	< 0.001**
GDS	2.9 (2.3)	2.7 (2.0)	2.2 (2.0)	2.8 (1.5)	3.3 (2.1)	3.4 (2.3)	3.4 (3.2)	2.3 (1.3)	2.8 (2.5)	0.898**
WMS-R Attention/Concentration	66.6 (12.2) (n = 124)^b^	69.3 (11.6) (n = 36)	59.3 (13.0) (n = 4)	61.3 (12.8) (n = 13)	63.1 (11.9) (n = 21)	57.3 (8.5) (n = 4)	69.2 (11.5) (n = 26)	61.2 (12.9) (n = 9)	74.5 (9.9) (n = 11)	0.013
WMS-R Delayed recall	47.0 (30.5) (n = 123)^c^	68.8 (17.9) (n = 36)	47.8 (48.6) (n = 4)	23.5 (21.2) (n = 13)	20.7 (19.3) (n = 20)	19.3 (7.8) (n = 4)	49.0 (29.4) (n = 26)	26.4 (27.9) (n = 9)	73.2 (14.8) (n = 11)	< 0.001
WMS-R Logical Memory II	10.1 (9.8) (n = 127)^d^	16.5 (7.7) (n = 36)	11.2 (15.0) (n = 5)	3.2 (5.5) (n = 13)	2.6 (6.0) (n = 22)	0.8 (1.3) (n = 5)	11.4 (9.2) (n = 26)	4.4 (6.3) (n = 9)	17.3 (9.4) (n = 11)	< 0.001
Odor identification scores	5.3 (2.5)	6.7 (2.2)	4.2 (2.3)	5.2 (1.7)	4.1 (2.3)	2.6 (0.9)	5.6 (2.6)	3.8 (2.3)	5.6 (2.3)	< 0.001
CSF Aβ1-42 pg/ml	940.5 (471.1)	1316.4 (379.3)	456 (118.3)	445.5 (80.9)	456.3 (116.7)	408.6 (139.8)	1084.1 (279.4)	1035.9 (380.2)	1267 (396.6)	–
CSF Aβ1-40 pg/ml	8554 (3398)	9489 (3099)	4866 (1574)	6722 (2436)	9506 (4368)	7558 (4937)	7325 (2735)	9681 (2348)	9995 (2693)	–
CSF Aβ42/40 ratio	0.136 (0.173)	0.148 (0.048)	0.099 (0.023)	0.072 (0.021)	0.057 (0.028)	0.217 (0.384)	0.216 (0.325)	0.113 (0.051)	0.133 (0.047)	–
CSF p-Tau pg/ml	69.59 (39.44)	38.45 (13.03)	27.73 (14.67)	90.46 (24.23)	92.16 (24.49)	48.32 (7.57)	100.80 (46.60)	95.56 (32.87)	39.52 (10.42)	–
CSF t-Tau pg/ml	709.7 (397.1)	483.4 (196.4)	489.4 (254.1)	507.6 (159.2)	1139.3 (391.4)	1103.2 (219.9)	447.1 (182.0)	1307.1 (329.9)	878.2 (81.1)	–
CSF p-Tau/Aβ1-42 ratio	0.104 (0.090)	0.031 (0.012)	0.057 (0.018)	0.209 (0.069)	0.218 (0.089)	0.136 (0.071)	0.098 (0.050)	0.102 (0.05)	0.033 (0.013)	–

Data are shown as mean (standard deviation) unless otherwise stated. *P*-values with no asterisk are for one-way analysis of variance (ANOVA).

**Kruskal-Wallis test.

*A(*+*)* amyloid-positive individuals with ATN classification; *A(−)* amyloid-negative; *T(*+*)* p-Tau positive; *T(−)* p-Tau negative; *N(*+*)* t-Tau positive; *N(−)* t-Tau negative; *aMCI* amnestic mild cognitive impairment; *AD* Alzheimer’s disease; *BMI* body mass index; *MMSE* Mini-Mental State Examination; *ADAS-cog J* Alzheimer-Disease Assessment Scale-Cognitive-Japanese version; *FAB* Frontal Assessment Battery; *GDS* 15-item Geriatric Depression Scale; *WMS-R* Wechsler Memory Scale-revised; *CSF* cerebrospinal fluid; *Aβ* amyloidβ; *p-Tau* Tau phosphorylated at threonine 181; *t-Tau* total Tau; *SNAP* suspected non-AD pathophysiology.

^a^Four participants did not consent.

^b^Eight participants did not consent.

^c^Nine participants did not consent.

^d^Five participants did not consent.

*P*-values were calculated for mean comparisons among the eight groups (Normal, AD continuum, and SNAP). The null hypothesis was that all the means of the eight groups were equal. We applied one-way ANOVA for normally distributed variables and the Kruskal–Wallis test for non-normally distributed variables.

The cutoff values for odor identification scores were determined through ROC analysis to distinguish among four disease stages (normal biomarkers, amyloidogenesis, p-Tau accumulation, AD neurodegeneration) consisting of seven distinct pathological conditions (A−/T−/N−, A+/T−/N−, A+/T+/N−, A+/T+/N+, A−/T+/N−, A−/T+/N+, and A−/T−/N+). The pathological interpretation of each category within the ATN classification is as follows: A−T−N− signifies normal biomarkers. A + T−N−, A + T + N−, A + T + N+, and A + T−N + denote the AD continuum, with the potential inclusion of suspected non-AD pathophysiology. (SNAP) in the A + T−N + category. Additionally, A−T + N−, A−T−N+, and A−T + N + may be construed as indicative of SNAP^12^ (Table 3, Supplementary Fig. S4).

**Table 3:** **Ability of the odor identification score to discriminate between categories of the A/T/N classification and corresponding cut-off values**.

Analysis No	Group 1	Versus Group 2	Comparative pathological stage	Mean (SD) (Group 1/Group 2)	*P*-value (t-test)	AUC	95% CI (De Long)	Cut-off	Specificity	Sensitivity
1	A−/T−/N−	A+/T−/N− A+/T+/N− A+/T+/N+	Normal vs. after amyloidogenesis	6.7(2.2)/4.5(2.1)	< 0.0001	0.761	0.658–0.864	5.5	0.676	0.659
2	A−/T−/N− A+/T−/N−	A+/T+/N− A+/T+/N+	Normal to amyloidogenesis vs. after p-Tau accumulation	6.3(2.3)/4.5(2.1)	0.0006	0.717	0.604–0.83	4.5	0.830	0.514
3	A−/T−/N− A+T−/N− A+/T+/N−	A+/T+/N+	Normal to p-Tau accumulation vs. AD neurodegeneration	6.1(2.2)/2.2(2.3)	0.0011	0.716	0.588–0.844	4.5	0.786	0.545
4	A−/T−/N−	A−/T+/N− A−/T+/N+	Normal vs. p-Tau accumulation without amyloidogenesis	6.7(2.2)/5.1(2.6)	0.0049	0.670	0.546–0.794	4.5	0.892	0.486
5	A−/T−/N−	A−/T+/N− A−/T+/N+ A−/T−/N+	Normal vs. SNAP	6.7(2.2)/5.2(2.5)	0.0050	0.653	0.538–0.768	4.5	0.892	0.429

The discriminative ability of odor identification scores according to the A/T/N classification was evaluated. Regardless of the presence or absence of neurodegeneration, the AUC for odor identification scores was high when amyloidogenesis occurred (Analysis 1). Analyses 2 and 3 were less sensitive, making it difficult to differentiate the processes leading to p-Tau  accumulation and neurodegenerative stages after amyloidogenesis. The AUC and sensitivity were low in the normal to p-Tau accumulation stage without amyloidogenesis regardless of the presence or absence of neurodegeneration (Analysis 4). After setting odor cut-off values for each A/T/N stage (A−/T−/N−, A+/T−/N−, A+/T+/N−, and A+/T+/N+), a post hoc power analysis was conducted; all stages met the criteria of a significance level of 5%, and a power of 80% at an AUC value ≥ 0.652. The results indicated that the sample size was sufficient to determine the cutoff value. Therefore, odor identification scores are excellent for differentiating amyloidogenesis but not p-Tau accumulation.

*A (*+*)* amyloid-positive individuals with A/T/N classification; *A (−)* amyloid-negative; *T (*+*)* p-Tau positive; *T (−)* p-Tau negative; *N (*+*)* t-Tau positive; *N (−)* t-Tau negative; *SD* standard deviation; *AUC* area under the curve; *CI* confidence interval; *p-Tau* phosphorylated Tau; *SNAP* suspected non-AD pathophysiology.

Next, we examined the correlations between the odor identification score and neuropsychological score or CSF biomarkers in four different stages based on the A/T/N classification (A−/T−/N−, A+/T−/N−, A+/T+/N−, and A+/T+/N+) and considering the presence or absence of ApoE4 (Table 4). We analyzed the correlation between odor identification scores and CSF biomarkers, adjusting for age, sex, ApoE4 status, and MMSE scores across three groups: A, B, and C. (Table 5: A: a group of the normal class and three AD continuum classes, B: a group of the normal class and two p-Tau accumulation classes without amyloidogenesis, C: a group of the normal class and three SNAP classes). Differences in the area under the curve (AUC) were analyzed using the bootstrap method with a resampling frequency of 20,000 to determine whether the odor identification score could replace each of the CSF biomarkers considered (Table 6, Supplementary Fig. S5).

**Table 4:** **Correlation of odor identification scores with neuropsychological test results and CSF biomarker levels in selected categories of the A/T/N classification (A−/T−/N−, A+/T−/N−, A+/T+/N−, and A+/T+/N+), both in the presence and absence of ApoE4**.

	Total		ApoE4(+)		ApoE4(−)	
	rho	*P*-value	rho	*P*-value	rho	*P*-value
Neuropsychological Test						
MMSE	**0.517**	** < 0.0001**	0.258	0.194	**0.425**	**0.0019**
ADAS-cog J	− **0.551**	**< 0.0001**	− 0.222	0.267	− **0.468**	**0.0006**
FAB	**0.509**	**< 0.0001**	0.001	0.996	**0.584**	**< 0.0001**
GDS	− 0.014	0.907	0.052	0.798	− 0.035	0.809
WMS-R Attention/Concentration score	**0.381**	**0.001**	0.175	0.404	**0.467**	**0.0007**
WMS-R Delayed recall score	**0.576**	**< 0.0001**	0.259	0.212	**0.526**	**0.0001**
WMS-R Logical Memory II score	**0.578**	**< 0.0001**	**0.386**	**0.047**	**0.464**	**0.0008**
CSF biomarkers						
Aβ40	0.027	0.8132	0.020	0.922	− 0.006	0.9685
Aβ42	**0.386**	**0.0005**	0.216	0.280	0.252	0.0739
Aβ42/40 ratio	**0.425**	**0.0001**	0.305	0.122	**0.317**	**0.0235**
p-Tau	− **0.373**	**0.0008**	− **0.387**	**0.046**	− 0.143	0.3172
t-Tau	− **0.229**	**0.0435**	− 0.285	0.150	− 0.088	0.5416
p-Tau /Aβ42 ratio	− **0.432**	**< 0.0001**	− **0.418**	**0.030**	− 0.250	0.0775
p-Tau/t-Tau ratio	− 0.129	0.2615	− 0.152	0.450	− 0.021	0.885

Significant correlations were found between odor identification scores and the main neuropsychological tests (MMSE, ADAS-cog J, FAB, Attention/Concentration, Delayed recall, and Logical memory II) using Spearman rank correlation and between olfactory performance and CSF markers (Aβ42, Aβ42/40 ratio, p-Tau, t-Tau, and p-Tau/Aβ42).

*A (*+*)* amyloid-positive individuals with A/T/N classification; *A (−)* amyloid-negative; *T (*+*)* p-Tau positive; *T (−)* p-Tau negative; *N (*+*)* t-Tau positive; *N (−)* t-Tau negative; *AUC* area under the curve; *CSF* cerebrospinal fluid; *MMSE* Mini-Mental State Examination; *WMS-R* Wechsler Memory Scale-revised; *ADAS-cog J* Alzheimer-Disease Assessment Scale-Cognitive-Japanese version.

Significant values are in [bold].

**Table 5:** **Correlation of corrected odor identification scores (adjusted for age, sex, ApoE4, and MMSE) with CSF biomarker levels: Spearman's rho of studentized residuals**.

CSF AD biomarkers	rho	*P*-value
A. Normal biomarkers and selected AD continuum group (A−/T−/N−, A+/T−/N−, A+/T+/N−, and A+/T+/N+)		
Aβ40	− 0.013	0.911
Aβ42	**0.230**	**0.043**
Aβ42/40 ratio	**0.292**	**0.010**
p-Tau	− 0.097	0.400
t-Tau	− 0.183	0.109
p-Tau/Aβ42 ratio*	− 0.206	0.070
p-Tau/t-Tau ratio*	0.042	0.716
B. Normal biomarkers and p-Tau accumulation without amyloidogenesis group (A−/T−/N−, A−/T+/N−, and A−/T+/N+)		
Aβ40	0.198	0.094
Aβ42	0.195	0.099
Aβ42/40 ratio	− 0.033	0.781
p-Tau	− 0.040	0.739
t-Tau	0.082	0.491
p-Tau/Aβ42 ratio	− 0.084	0.479
p-Tau/t-Tau ratio	− 0.034	0.774
C. Normal biomarkers and SNAP group (A−/T−/N−, A−/T+/N−, A−/T+/N+, and A−/T−/N+)		
Aβ40	0.123	0.261
Aβ42	0.130	0.237
Aβ42/40 ratio	− 0.024	0.831
p-Tau	− 0.036	0.742
t-Tau	0.032	0.769
p-Tau/Aβ42 ratio	− 0.048	0.661
p-Tau/t-Tau ratio	− 0.041	0.712

Weak correlations were found between corrected odor identification scores and CSF biomarkers (Aβ42 and Aβ42/40 ratio) in the group characterized by normal biomarkers and selected AD continuum group (A).

No correlations were found between corrected scores for odor identification and CSF biomarkers (Aβ42 and Aβ42/40 ratio) in the group characterized by normal biomarkers and p-Tau accumulation without amyloidogenesis (B), as well as in the group characterized by normal biomarkers and SNAP (C).

*CSF* cerebrospinal fluid; *AD* Alzheimer’s disease; *SNAP* suspected non-AD pathophysiology.

Significant values are in [bold].

*Logarithmic transformation.

**Table 6:** **Differences in AUC values of the odor identification score and CSF biomarker levels using the bootstrap method.**

Biomarker	AUC	*P*-value (bootstrap)
Aβ42	0.825	0.455
p-Tau	0.721	0.274
t-Tau	0.721	0.317
Aβ42/Aβ40	0.777	0.898
p-Tau/Aβ42	0.836	0.315
Odor identification scores	0.785	–

Differences in AUC values of the odor identification scores and CSF biomarkers were examined using bootstrap sampling. The sampling frequency was 20,000. Receiver operating characteristic (ROC) analysis was used to discriminate between normal + aMCI and AD patients. We examined the difference in the AUC between the odor identification scores and each biomarker using the bootstrap method to determine whether the odor identification scores can be used as a CSF biomarker for AD diagnosis. AUCs for the odor identification scores  were not significantly different from those for Aβ42, p-Tau, t-Tau, the Aβ42/Aβ40 ratio, and the p-Tau/Aβ42 ratio; hence, the odor identification scores did not differ significantly from the alternative markers. Therefore, the odor identification scores may serve as a potential surrogate marker.

*AD* Alzheimer’s disease; *AUC* area under the curve; *CSF* cerebrospinal fluid.

Finally, we conducted logistic regression on 12 olfactory species for the odor identification score (Standard Version) and identified the optimal combination with the minimum Akaike Information Criterion (AIC) to detect amyloidogenesis in individuals diagnosed with AD (Table 7. The effectiveness of this combination was compared to that of the standard combination using ROC analysis (Tables 7 and 8, Supplementary Fig. S6).

**Table 7:** **Optimal odor identification score combination from standard version for discriminating normal biomarkers and AD continuum individuals.**

Variances	Estimate	Standard error	z value	Pr ( >|z|)
Analysis A. Coefficients of logistic regression model for the standard version (12 items)				
(Intercept)	4.172	1.589	2.625	0.0087**
A (India ink)	− 1.350	0.702	− 1.924	0.0544*
B (wood)	− 1.655	0.708	− 2.336	0.0195*
C (perfume)	− 1.000	0.708	− 1.414	0.1575
D (menthol)	0.651	0.869	0.750	0.4534
E (Japanese orange)	0.789	0.865	0.911	0.3622
F (curry)	− 2.125	1.461	− 1.455	0.1457
G (gas leak odor)	− 0.416	0.671	− 0.620	0.5354
H (rose)	− 1.204	0.727	− 1.657	0.0975*
I (hinoki (Japanese cypress wood))	0.418	0.781	0.535	0.5924
J (sweaty socks)	− 1.242	0.654	− 1.899	0.0576*
K (condensed milk)	− 1.016	0.747	− 1.361	0.1737
L (roasted garlic)	0.606	0.833	0.727	0.4671
AIC	–	–	–	98.57
Analysis B. Coefficients of the best logistic regression model for the selected version (4 items)				
(Intercept)	3.777	1.244	3.035	0.0024**
A (India ink)	− 1.254	0.602	− 2.082	0.0373*
B (wood)	− 1.960	0.598	− 3.277	0.0011**
F (curry)	− 1.885	1.203	− 1.567	0.1170
J (sweaty socks)	− 1.197	0.604	− 1.980	0.0477*
AIC	–	–	–	90.189

The table for Analysis A presents the estimated coefficients (Estimate), their respective standard errors, z-values, and *p*-values for various variables in a logistic regression model. Each variable (A, B, C, etc.) represents different odors or predictors analyzed within the model. The estimates signify the magnitude and direction of the effect each variable has on the outcome being studied. The standard errors provide a measure of the variability or uncertainty around these estimates. The z-values and *p*-values indicate the statistical significance of each variable; lower *p*-values generally suggest stronger evidence against the null hypothesis, implying a more significant impact of the variable on the outcome. Lastly, the AIC (Akaike Information Criterion) with a value of 98.57 serves as a measure of model fit, where lower values indicate a better fit of the model to the data.

The table for Analysis B displays estimates, standard errors, z-values, and *p*-values for variables in a logistic regression model. Variables A, B, F, and J show statistically significant relationships indicated by their associated p-values (* and **). The AIC value of 90.189 assesses model fit. AIC is lower for the selected type compared to the standard version, indicating superior odor identification in the selected version.

*AIC* Akaike Information Criterion.

*T test; **Wilcoxon rank sum test.

**Table 8:** **Comparison of odor identification scores between the standard version and the selected version among individuals with normal biomarkers and those in the AD continuum**.

	Group 1	Group 2	Mean (SD) (Group 1/Group 2)	*P*-value (mean)	AUC	95% CI (De Long)	Cut-off	Specificity	Sensitivity	*P*-value (AUC)
Standard version (12 items)	A − T − N −	A + T − N−,A + T + N−, A + T + N +	6.7(2.2)/4.5(2.1)	< .0001*	0.761	0.658–0.864	5.5	0.676	0.659	0.265
Selected version (4 items***)	A − T − N −	A + T − N− ,A + T + N−, A + T + N +	2.8(0.7)/1.7(0.9)	< .0001**	0.809	0.718–0.900	2.5	0.676	0.854	

This analysis compared a 12-item standard version with a 4-item selected version in an odor identification test between individuals with normal biomarkers and those within the selected Alzheimer's disease continuum. The mean values for both groups showed significant differences between the standard (Group 1: 6.7, Group 2: 4.5) and selected versions (Group 1: 2.8, Group 2: 1.7). Both versions exhibited statistical significance (*p* < .0001) in this comparison. The selected version demonstrated an improved area under the curve (AUC) of 0.809 (95% CI: 0.718–0.900) compared to the standard version's AUC of 0.761 (95% CI: 0.658–0.864). Notably, the selected version displayed a lower cutoff value (2.5) with higher sensitivity (0.854), indicating potential advantages over the standard version in diagnostic performance.

*SD* standard deviation; *AUC* area under the curve.

*T test; **Wilcoxon rank sum test, *** A: India ink, B: wood, F: curry, J: sweaty socks.

## Results

**Figure 1:**
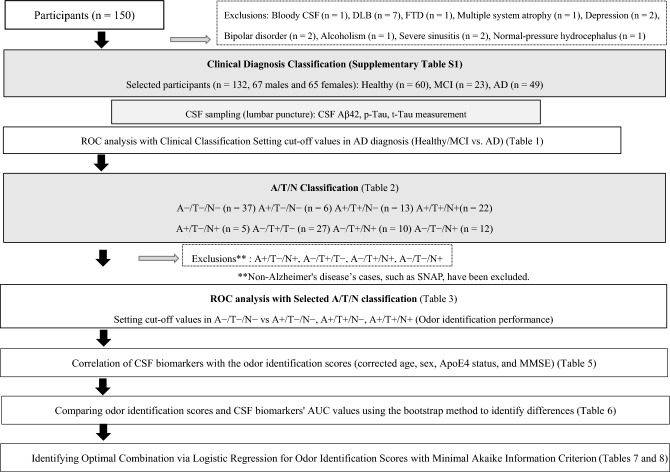
**Flowchart of participants from examination to A/T/N classification and evaluation of olfactory discrimination**. *CSF* cerebrospinal fluid; *DLB* diffuse Lewy body disease; *FTD* frontotemporal dementia; *MCI* mild cognitive impairment; *AD* Alzheimer's disease; *ROC* receiver operating characteristic; *Aβ* amyloid-β; *p-Tau* phosphorylated Tau; *t-Tau* total Tau; *A*+ amyloid-positive individuals; *A− *amyloid-negative individuals; *T*+ phosphorylated Tau-positive individuals; *T− *phosphorylated Tau-negative individuals; *T*+ total Tau-positive individuals; *T− *total Tau-negative individuals; *SNAP* suspected non-Alzheimer’s disease pathophysiology; *ApoE* apolipoprotein E; *MMSE* Mini-Mental State Examination; *AUC* area under the curve.

A total of 132 patients were analyzed in this study, including 49 patients with AD [mean age: 71.7 ± 7.9 years, age range: 50–79 years, 34 men (69%) and 15 women (31%)], 23 patients with amnestic MCI (aMCI) [mean age: 72.7 ± 6.9 years, age range: 56–80 years, 11 men (48%) and 12 women (52%)], and 60 healthy controls [mean age: 70.2 ± 7.3 years, age range: 41–80 years, 35 men (60%) and 25 women (40%)] (Fig. 1, Supplementary Table ST1).The study workflow is illustrated in Fig. 1.

The average age of the 132 participants was 71.2 ± 7.5 years. Sixty-seven were men (50.8%) and 65 were women (49.2%). The years spent in formal education averaged 14.2 ± 2.2, and there were no significant differences observed among the normal, aMCI, and AD groups. The overall number of ApoE4 carriers was 44 individuals (33.6%), with a significantly higher prevalence in the AD group (32 individuals; 65.3%). The MCI group included seven carriers (30.4%), and the healthy control group included five (8.5%). The odor identification score had an average value of 5.3 ± 2.5 points, with the healthy control and MCI groups scoring 6.2 ± 2.1 and 6.1 ± 2.4 points, respectively. In contrast, participants with AD scored 3.8 ± 2.1, indicating a significant decline compared to the other two groups (Supplementary Table ST1).

Participant attributes based on clinical diagnosis classification (Supplementary Table ST1) and A/T/N classification were compared (Table 2) using one-way ANOVA or Kruskal–Wallis tests. Significant differences were observed in MMSE, ADAS-cog J, FAB, WMS-R A/C, DR, LM-II, and odor identification scores, as well as in CSF Aβ1-42, p-Tau181, and t-Tau levels, and the Aβ42/40 ratio (all *P* < 0.001). Additionally, significant differences between the healthy control and the AD group were noted for all these variables. Correlations between the values from the aMCI and AD groups were found for MMSE, ADAS-cog J, FAB, WMS-R A/C, DR, LM-II, and odor identification scores, as well as for CSF Aβ1-42 and t-Tau levels (Supplementary Table ST1).

ROC analysis was performed to further evaluate the discriminatory power of the odor identification score in differentiating between the three experimental groups. The AUC for the odor identification score (0.785) was similar to that of Aβ42/Aβ40 (0.777; Table 1). The resulting cutoff values were 658.48 pg/ml (AUC: 0.825, sensitivity: 71.4%, specificity: 85.5%) for Aβ42, 58.37 pg/ml (AUC: 0.721, sensitivity: 79.6%, specificity: 60.2%) for p-Tau and 756.6 pg/ml (AUC: 0.721, sensitivity: 63.3%, specificity: 77.1%) for t-Tau (Table 1, Supplementary Fig. S2). We categorized participants based on the A/T/N classification using these specific cutoff values, resulting in the distribution outlined in Table 2. The normal AD biomarker group (A−T−N−) showed significant declines in multiple neuropsychological assessments compared to the AD continuum groups (A+/T−/N−, A+/T+/N−, and A+/T+/N+), worsening with higher ATN stages. Specifically, significant differences were observed in MMSE, ADAS, FAB, DR, LM-II, and odor identification (all *P* < 0.001). This result indicates that the A/T/N classification system accurately reflects clinical progression, as depicted in Supplementary Fig. S3. Multiple comparisons were conducted across the eight stages of the A/T/N classification, focusing on p-values indicating significance at the 5% and 10% levels (Supplementary Table ST2). In some cases, even though we initially observed differences at a level of significance of 5% in the ANOVA or Kruskal–Wallis test (Table 2), subsequent multiple comparisons did not show any differences at this significance level. A summary of the statistical comparison between different categories of the A/T/N classification is shown in Supplementary Table ST2.

Post-hoc analysis revealed significant differences between the group with normal AD biomarker levels (A−/T−/N−, group 1) and the AD continuum group (A+/T−/N−, group 2; A+/T+/N−, group 3; A+/T+/N+, group 4; and A+/T−/N+, group 5) for MMSE [(1) vs. (3), *P* = 0.043], ADAS-cog J [(1) vs. (3),* P* = 0.010; (1) vs. (4), *P* = 0.010; (1) vs. (5), *P* = 0.025], delayed recall [(1) vs. (3), *P* = 0.004; (1) vs. (4), *P* < 0.001], and logical memory II [(1) vs. (3),* P* = 0.008; (1) vs. (4), *P* < 0.001]. However, no statistically significant differences at the 5% significance level were found for the odor identification score [(1) vs. (4),* P* = 0.060; (1) vs. (5),* P* = 0.069] or FAB [(1) vs. (3),* P* = 0.080]. In the comparison between the group with normal biomarker levels and the SNAP group (A−/T + N−, group 6; A−/T+/N+, group 7; and A−/T−/N+, group 8), significant differences were evident for MMSE [(1) vs. (7), *P* = 0.038] and DR [(1) vs. (7),* P* = 0.039]. Furthermore, significant differences were observed between the AD continuum and the SNAP groups for ADAS-cog J [(4) vs. (8),* P* = 0.028], DR [(3) vs. (8),* P* = 0.035; (4) vs. (8),* P* = 0.007], and LM-II [(4) vs. (8),* P* = 0.011] (Supplementary Table ST2).

Furthermore, we assessed the discriminatory ability of the odor identification score at each A/T/N pathological stage (Table 3). The odor identification score had a higher AUC (0.761, sensitivity = 0.659, specificity = 0.676) during amyloidogenesis compared to changes in biomarker levels (Analysis No. 1), irrespective of the presence or absence of neurodegeneration. However, during the p-Tau accumulation (Analysis No. 2, AUC = 0.717, sensitivity = 0.514, specificity = 0.860) and neurodegeneration stages (Analysis No. 3, AUC = 0.716, sensitivity = 0.545, specificity = 0.786), the sensitivity of the odor identification score was low, making the distinction between p-Tau accumulation and neurodegeneration after amyloidogenesis challenging. In addition, the AUC (0.670) and sensitivity (0.486) were low in the comparison of individuals from the normal to the p-Tau accumulation stages with or without neurodegeneration (Analysis No. 4). Therefore, the odor identification score offers superior performance for the identification of individuals in the amyloidogenesis stage, but it is unsuitable for discriminating between the p-Tau accumulation and neurodegeneration stages, or for identifying p-Tau accumulation without amyloidogenesis and SNAP. The details of the findings are presented in Table 3 and Supplementary Fig. S4. A post-hoc power analysis showed that all analyses had a significance level of *P* < 0.05 and a power above 0.8. The power values for the comparisons described above were 0.996, 0.966, 0.925, 0.837, and 0.809, respectively. As statistically significant ROC curves were obtained for the biomarker of interest, as illustrated in Supplementary Fig. S4, we were able to determine the cutoff value even with our current sample size. However, for a comprehensive evaluation of the estimated cutoff value, it is imperative to apply the same analysis to an independent set of samples. This step will confirm the adequacy of the sample size for the intended purpose.

Further analyses were performed to examine the association between the odor identification score, the neuropsychological test scores, and CSF biomarker levels in individuals with and without the ApoE4 isoform (Table 4). The results showed a significant correlation between the odor identification score and the scores of the neuropsychological tests (MMSE, *P* < 0.0001; ADAS-cog, *P* < 0.0001; FAB, *P* < 0.0001; DR, *P* < 0.0001; and LM-II, *P* < 0.0001), independently of ApoE4 status. Significant correlations were observed between the odor identification score and the levels of CSF biomarkers that are valid for AD diagnosis (CSF Aβ42, *P* = 0.0005; Aβ42/40, *P* = 0.0001; p-Tau/Aβ42 ratio, *P* < 0.0001; Table 4). Further analysis showed that the odor identification score had a weak correlation with Aβ42 levels in CSF and with the Aβ42/40 ratio, after adjusting for age, sex, ApoE4 status, and performance in the MMSE test in the normal biomarker and selected AD continuum group (Spearman’s rho = 0.230, *P* = 0.043 and rho = 0.292, *P* = 0.010, respectively; Table 5A). The odor identification score displayed no correlation with any AD biomarkers in the CSF for either the group classified as the normal biomarkers and p-Tau accumulation without amyloidogenesis group (Table 5B), or in the normal and SNAP group (Table 5C).

Finally, we investigated whether the odor identification score could be used as a substitute for CSF biomarkers in AD diagnosis. The AUC for the odor identification score and for several CSF biomarkers were compared using the bootstrap method. No significant differences were observed in AUC between the odor identification score and any of the CSF biomarkers, including Aβ42 (*P* = 0.455) and the Aβ42/Aβ40 (*P* = 0.898) and p-Tau/Aβ42 ratios (*P* = 0.315). These findings demonstrate that odor identification score has the potential to serve as a surrogate marker for these CSF biomarkers in AD diagnosis and can discriminate amyloid changes in AD. The findings are summarized in Table 6, and ROC curves are presented in Supplementary Fig. S5. Logistic regression on the overall odor identification score and on each of the subscores corresponding to 12 individual odors revealed greater contributions from four odors: India ink, wood, curry, and sweaty socks. Compared to the entire array of 12 odors, the use of this specific combination of four resulted in an improved Akaike's Information Criterion (AIC) value and in a higher sensitivity (0.854 > 0.659) and AUC value (0.809 > 0.761) according to the ROC analysis (Tables 7 and 8, Supplementary Fig. S6).

## Discussion

Odor identification tests have gained attention as a potential screening tool for the early stages of AD. Approximately 85–90% of patients with AD exhibit olfactory impairment, with a reported sensitivity and specificity of approximately 85% compared to healthy individuals^21^. Individuals with severe olfactory impairment, even with normal cognitive function, have a higher risk of developing MCI^22^. Moreover, individuals with MCI that exhibit olfactory impairment are more likely to progress to advanced cognitive dysfunction and develop AD^23^. A reduced odor identification score indicates symptom progression even in cases of subjective cognitive decline^24,25^. Furthermore, the odor identification score has been reported to possess superior performance when distinguishing between AD and healthy participants compared to between stages of AD^26^. The odor identification score excelled in differentiating between healthy controls and prodromal AD (AUC = 0.908) but was not as effective in distinguishing between prodromal AD and AD dementia (AUC = 0.773)^27^. These findings show that odor identification scores are highly sensitive in the early stages of AD but may not be as suitable for monitoring disease progression^27^. High scores on the 40-item University of Pennsylvania Smell Identification Test are typically linked to a negative amyloid PET scan result^28^. Impaired odor identification was observed in older adults with elevated cortical amyloid levels, suggesting the potential of the odor identification score for detecting preclinical AD in cognitively normal individuals^29^.

However, meta-analysis reveals minimal associations between odor identification and PET or CSF biomarkers for AD among older adults^30^. Moreover, impaired odor identification within the AD spectrum may be a result of neurodegeneration rather than a direct impact of Aβ or p-Tau burden^31^.

### Association between the odor identification score and Aβ deposition

In both mouse models and in humans, evidence suggests that Aβ protein deposition in the olfactory system is associated with olfactory dysfunction and neuronal damage. Mouse models such as Tg2576 mice show Aβ deposition in the olfactory bulb (OB) before cognitive impairment^32–34^. Similarly, Amyloid precursor protein/Presenilin1 mice exhibit Aβ deposition throughout the olfactory circuit^35^. Furthermore, 5 × Familial AD mice, a model for AD pathology, display high levels of Aβ accumulation in the olfactory sensory network^36^. In humans, amyloid deposition directly affects olfactory regions in the brain, leading to olfactory impairment^37^. Autopsy studies on 536 cases examining OB neuropathology in AD cases showed Aβ accumulation in the OB starting from stage 0 of Tahl Aβ phase, progressing as they advance to stage 5^37^.

Soluble Aβ has been identified in nasal secretions of patients with AD, and high levels of nasal oligomers have been linked to AD progression within three years^38^. This suggests that Aβ oligomers may impair the olfactory nerve even before Aβ accumulation is observed in the OB. Nevertheless, odor identification scores in AD may not be directly related to Aβ burden, as indicated by reports of no difference in odor discrimination scores between Pittsburgh compound B (PiB)-positive and PiB-negative patients with MCI^39^. Aβ pathology reaches a plateau early during the symptomatic phase of the disease and does not correlate well with clinical features or with AD severity^40^.

### Association between odor identification score and p-Tau and other Tau accumulation

Alternatively, olfactory impairment in AD may be linked to p-Tau accumulation rather than the presence of Aβ in the OB. NFTs, a pathological hallmark of AD and neurodegeneration^41^, are frequently observed in the OB^42,43^. The distribution of NFTs in AD is classified according to the Braak classification into stages I–VI^40^. In the preclinical stage of AD, p-Tau accumulation begins in the entorhinal cortex (Braak stages I and II). This p-Tau accumulation leads to olfactory impairment associated with NFT accumulation in the entorhinal cortex and CA1 region of the hippocampus^44^.

Tau is more likely to accumulate in the OB as AD progresses, increasing with the Braak stage, leading to a worsening in olfactory dysfunction. Tau is believed to be the primary protein involved in the pathogenesis of olfactory impairment in AD. However, it has been reported that while Aβ pathology in the OB aligns with AD manifestation, Tau pathology in the OB is universally present in older adults and does not serve as an indicator of AD severity^37^. This suggests that Aβ deposition in the OB may serve as an early marker of AD onset. Olfactory impairment is correlated with CSF t-Tau/Aβ1-42 ratio, p-Tau/Aβ1-42 ratio, and t-Tau levels, but not with Aβ1-42 levels alone, except in ApoE ε4 carriers^45^. However, in the analysis of CSF biomarkers, no significant associations were observed between the odor identification score and CSF p-Tau or Aβ1-42 levels^30^.

### Association between odor identification score and neurodegeneration

Baek et al. analyzed olfactory function according to the A/T/N classification using PET and found a correlation between declining olfactory function and decreased neuropsychological test scores. However, after adjusting for cognitive function, olfactory impairment was not correlated with Aβ or Tau but rather with the volume of the temporal lobe cortex^31^. These findings suggest that olfactory impairment is primarily influenced by neurodegeneration rather than by amyloid or Tau pathologies.

CSF biomarkers reportedly exhibit a faster rate of change in AD compared to PET imaging^46^. PET imaging may be more useful to assess a later stage of the disease compared to the A/T/N classification based on CSF biomarkers, likely influenced by neurodegeneration.

### Our findings, odor identification impairments, and CSF biomarkers

Our institution has implemented a standardized CSF sample collection method for A/T/N classification to prevent potential misclassification of healthy individuals as patients with AD due to inappropriately measured Aβ42 levels. Additionally, the A/T/N classification system excludes SNAP cases, enhancing AD diagnostic accuracy. By excluding SNAP cases and improving A/T/N classification accuracy, we found that olfactory ability correlated slightly with the Aβ42/40 ratio but not with p-Tau after narrowing down the participant population. The A/T/N classification system based on CSF biomarkers captures pathological brain changes at an earlier stage compared to PET imaging. Therefore, odor identification impairments precede stage 0 of the Tahl Aβ phase, characterized by Aβ42 accumulation in the OB^37^. Olfactory impairment also occurs shortly after the onset of Aβ42 accumulation in the cerebral cortex.

Our results suggest that olfactory identification tests are effective in distinguishing preclinical AD during the transition from normal to amyloidogenesis. We also confirm the decrease in olfactory identification ability as the disease progresses, from A + to T + and N +. This indicates that the olfactory identification score can serve as a reliable, non-invasive, and cost-effective marker for improving the accuracy of screening for very early stage of AD. Moreover our clinical classification study found a correlation between olfactory impairment and several cognitive test scores, including the ADAS-cog J, DR, LM-II, and FAB. This demonstrates the involvement of the frontal lobe beyond the olfactory cortex and limbic system. Early Aβ42 accumulation in the default mode network (DMN), which includes the orbitofrontal cortex (OFC) and posterior cingulate cortex (PCC)^47^, likely impairs olfactory function by damaging these areas. The OFC serves as the endpoint of the olfactory pathway, whereas the PCC is functionally connected to the anterior cingulate cortex and hippocampus/intraorbital cortex^47^. Functional MRI studies have indicated that reduced task-related activation of the olfactory network is associated with decreased task-related inhibition of the DMN, which coincides with Aβ plaque deposition in the frontal lobe^48,49^. Therefore, Aβ may impair the DMN, gradually decreasing the olfactory identification score before p-Tau accumulates in the entorhinal cortex.

### Improved discriminative ability of a 4-item odor identification score for accumulated Aβ42 in the orbitofrontal cortex

As shown in Table 8 and Supplementary Fig. S6, the use of a combination of four odors (India ink, wood, curry, and sweaty socks) identified AD amyloidogenesis with a higher sensitivity, suggesting its potential for adoption in a clinical setting. Recent studies suggest that odor discrimination, particularly that involved in distinguishing between types of smells, primarily occurs in the orbitofrontal cortex (OFC), whereas the piriform cortex (PirC) and amygdala show less intricate discrimination^50^. The accumulation of Aβ42 in the OFC suggests that variations in the specific regions within the OFC may determine the types of impaired olfactory components.

### Limitations, challenges, and potential clinical application of odor identification score to other neurodegenerative disorders

This study has certain limitations. The exclusive focus on a Japanese population raises concerns about generalizability due to potential regional variations influenced by environmental and dietary factors. Excluding SNAP cases (including those of Parkinson's disease [PD]) affects the diagnostic accuracy of AD, posing challenges in real-world clinical settings. While the odor identification score could indicate early AD stages, larger-scale studies encompassing diverse ethnic backgrounds are required. Excluding patients with severe dementia due to challenges in advanced assessments like ADAS-cog J or WMS-R might introduce bias. This exclusion potentially constrains the study's relevance to those with mild to moderate dementia. Nevertheless, it is noteworthy that both odor identification score measurements and CSF biomarker analyses were conducted in patients with severe dementia, aiding in bias mitigation.

A significant limitation is that not all participants had MIBG or DAT scans. Olfactory dysfunction is a common early sign of both DLB/PD and AD, the most prevalent neurodegenerative diseases in older adults^51^. Out of the 132 participants, only 43.9% underwent these scans, possibly concealing latent DLB or PD cases. Nevertheless, participants without these scans exhibited posterior cerebral hypoperfusion on SPECT, leading to their exclusion from the study. Low odor identification scores can also be observed in frontotemporal dementia FTD^51^. Thus, the study emphasizes the need for a comprehensive approach, considering clinical symptoms, brain MRI, and specialized tests such as MIBG and DAT scans for accurate diagnosis^52^.

Our data challenges conventional beliefs, suggesting CSF Aβ42 levels alone have higher specificity than the Aβ42/40 ratio. This ratio is essential for differentiating AD and non-AD cases identified by amyloid PET using CSF biomarkers. Notably, Aβ42 alone exhibits better predictive performance for AD onset in patients with MCI^8^. Our study also demonstrates that the odor identification score could be used as a substitute for CSF biomarkers, with its associated AUC being almost equivalent to that observed for Aβ42/Aβ40. However, it is not a direct determinant for future anti-amyloid therapy. Positive scores necessitate confirmation through CSF biomarkers or PET scans for a definitive diagnosis.

Nevertheless, the score is a very useful and cost-effective screening tool^51^. Conversely, in specific conditions such as progressive supranuclear palsy or corticobasal degeneration, low odor identification scores are not typically observed^52^, making the odor identification score useful in ruling out these conditions.

## Conclusion

The odor identification score offered a superior performance in the discrimination of amyloidogenesis with and without neurodegeneration but not in that between p-Tau accumulation and neurodegeneration. These findings indicate that the odor identification score could be useful for differentiating amyloid changes according to the A/T/N classification. Thus, the odor identification score may be a useful diagnostic marker for preclinical AD and may be a suitable screening tool for disease-modifying therapies.

### Supplementary Information

Supplementary Information.

## Data Availability

The corresponding author will make the data supporting the findings of this study available upon request.
