# Supplementary material for: Odor identification score as an alternative method for early identification of amyloidogenesis in Alzheimer’s disease
Source: Sci Rep. 2024 Feb 26;14:4658. doi: 10.1038/s41598-024-54322-3 (PMC10897211; doi:10.1038/s41598-024-54322-3)
Supplement: Supplementary file 1 — Supplementary Information. [file 41598_2024_54322_MOESM1_ESM.docx]

**Supplementary materials**

**Odor identification score as an alternative method**

**for early identification of amyloidogenesis**

**in Alzheimer’s disease**

Yukifusa Igeta^1,2^, Isao Hemmi^3^, Kohei Yuyama^4^, and Yasuyoshi Ouchi^1,2^

**Supplementary Table ST1. Characteristics of study population by clinical diagnosis classification**

| **Variables** | **Total** | **Control** | **aMCI** | **AD Dementia** | ***P*-value**  (ANOVA,  Kruskal Wallis, χ2) |  | ***P*-value**  (Multiple comparison) |  |
| --- | --- | --- | --- | --- | --- | --- | --- | --- |
| N | 132 | 60 | 23 | 49 |  | **Control vs aMCI** | **Control vs AD** | **aMCI vs**  **AD** |
| A (+), n (%) | 46(34.8) | 3(5.0) | 9(39.1) | 34(69.4) | **<0.001*** |  |  |  |
| Age (years) | 71.2(7.5) | 70.2(7.3) | 72.7(6.9) | 71.7(7.9) | 0.163** | 0.267 | 0.324 | 0.910 |
| Male (%) | 67/132(51) | 35/60(58) | 11/23(48) | 21/49(43) | 0.262* |  |  |  |
| Education, years | 14.4(2.2) | 14.6(2.1) | 14.6(2.3) | 14.0(2.4) | 0.430** |  |  |  |
| ApoE4 carrier number (%) | 44/131(34) | 5/59(9) | 7/23(30) | 32/49(65) | **<0.001*** |  |  |  |
| BMI | 22.2(3.7) | 23.5(3.6) | 21.4(3.2) | 21.1(3.7) | **0.005**** | 0.149 | 0.008 | 0.895 |
| MMSE score | 26.5(3.9) | 28.9(1.1) | 28.0(1.8 | 22.7(3.8) | **<0.001**** | **<0.001** | **0.031** | **0.031** |
| ADAS-cog J score | 7.3(5.8) (n=128)^a^ | 3.6(2.0) (n=59) | 5.5(2.2) (n=23) | 12.8(6.1) (n=46) | **<0.001**** | **0.048** | **<0.001** | **<0.001** |
| FAB score | 15.1(2.8) | 16.6(1.5) | 15.7(2.1) | 12.9(2.9) | **<0.001**** | 0.219 | <0.001 | 0.002 |
| GDS score | 2.9(2.3) | 2.8(2.5) | 3.0(2.4) | 3.1(1.8) | 0.341** |  |  |  |
| WMS-R Attention/Concentration score | 66.6(12.2) (n=124) ^b^ | 71.5(10.4) (n=60) | 67.9(13.1) (n=23) | 58.6(10.1) (n=41) | **<0.001** | 0.370 | **<0.001** | **0.003** |
| WMS-R  Delayed recall score | 47.0(30.5) (n=123) ^c^ | 71.6(17.0) (n=60) | 41.1(19.5) (n=23) | 13.4(12.2) (n=40) | **<0.001**** | **<0.001** | **<0.001** | **0.002** |
| WMS-R  Logical Memory II score | 10.1(9.8) (n=127) ^d^ | 18.2(7.0) (n=60) | 6.3(6.9) (n=23) | 1.0(2.4) (n=44) | **<0.001**** | **<0.001** | **<0.001** | **0.025** |
| Odor identification score | 5.3(2.5) | 6.2(2.1) | 6.1(2.4) | 3.8(2.1) | **<0.001** | 0.980 | **<0.001** | **<0.001** |
| CSF Aβ1-42 pg/ml | 940.5(471.1) | 1195.6(384.0) | 960.2(529.9) | 618.0(329.8) | **<0.001**** | 0.064 | **<0.001** | **0.020** |
| CSF Aβ1-40 pg/ml | 8554(3398) | 8677(3303) | 8968(2553) | 8209(3858) | 0.286** |  |  |  |
| CSF Aβ42/40 ratio | 0.1355(0.173) | 0.1723(0.221) | 0.1128(0.068) | 0.1010(0.127) | **<0.001**** | **0.008** | **<0.001** | 0.275 |
| CSF p-Tau181 pg/ml | 69.59(39.44) | 58.32(42.73) | 70.39(36.27) | 83.02(32.49) | **<0.001**** | 0.148 | **<0.001** | 0.261 |
| CSF t-Tau pg/ml | 709.7(397.1) | 568.8(259.5) | 623.6(244.3) | 922.8(495.6) | **<0.001** | 0.811 | **<0.001** | 0.004 |

All values are mean (standard deviation) unless otherwise stated. We applied one-way analysis of variance (ANOVA) for normally distributed variables and the Kruskal Wallis (KW) test for non-normally distributed variables. Further multivariate analysis was conducted among the control, aMCI, and AD dementia groups. For multiple comparisons as post hoc tests, we conducted the Tukey-Kramer test after ANOVA, and the Scheffe test after the Kruskal -Wallis test. Significant differences with p-values less than 5% are highlighted in bold. The P-value was used to assess whether the differences in means observed between different groups were statistically significant. P-values with no asterisk are for one-way ANOVA. *chi-squared test, **Kruskal Wallis test.

*aMCI* amnestic mild cognitive impairment; *AD* Alzheimer's disease; *ANOVA* analysis of variance; *A (+)* amyloid-positive individuals with ATN classification; *n* number; *ApoE4* Apolipoprotein E4; *BMI* body mass index; *MMSE* Mini-Mental State Examination; *ADAS-cog J* Alzheimer-Disease Assessment Scale-Cognitive-Japanese version; *FAB* Frontal Assessment Battery; *GDS15* 15-item Geriatric Depression Scale; *WMS-R* Wechsler Memory Scale-revised; *CSF* cerebrospinal fluid; *Aβ* amyloid-β; *p-Tau181* Tau phosphorylated at threonine 181; *t-Tau* total Tau.

^a^ Four participants did not consent. ^b^ Eight participants did not consent. ^c^ Nine participants did not consent. ^d^ Five participants did not consent.

**Supplementary Table ST2. Statistical determination of *P*-values for study population characteristics by A/T/N classification using multiple analytical approaches**

|  |  |  | ***P*-value** |  |  |  |
| --- | --- | --- | --- | --- | --- | --- |
| BMI | **(4) vs (6)**  **0.045** | (3) vs (6)  0.068 |  |  |  |  |
| **MMSE** | **(1) vs (3)**  **0.043** | **(1) vs (4)**  **0.002** | (1) vs (5)  0.071 | **(1) vs (7)**  **0.038** |  |  |
| ADAS-cog J | (1) vs (2)  0.098 | (1) **vs** (3)  **0.010** | (1) **vs** (4)  **<0.001** | **(1) vs (5)**  **0.025** | **(4) vs (8)**  **0.028** | (5) vs (8)  0.083 |
| FAB | (1) vs (3)  0.080 |  |  |  |  |  |
| WMS-R  Delayed recall | **(1) vs (3)**  **0.004** | **(1) vs (4)**  **<0.001** | **(1) vs (7)**  **0.039** | **(3) vs (8)**  **0.035** | **(4) vs (8)**  **0.007** |  |
| WMS-R  Logical Memory II | **(1) vs (3)**  **0.008** | **(1) vs (4)**  **<0.001** | (1) vs (7)  0.087 | (3) vs (8)  0.074 | (4) vs (6)  0.096 | **(4) vs (8)**  **0.011** |
| Odor identification scores | (1) vs (4)  0.060 | (1) vs (5)  0.069 |  |  |  |  |

In our statistical analysis involving multiple comparisons within the A/T/N classification, we conducted post hoc tests using the Tukey-Kramer test following ANOVA, and the Scheffe test following the Kruskal Wallis test. We did not find any significant differences below a 5% threshold in the odor identification scores. However, in some instances, initial observations of a 5% significance level in the ANOVA or Kruskal Wallis test (Table 2) were not upheld in subsequent multiple comparisons. To emphasize specific group comparisons within the A/T/N classification demonstrating differences, we reported pairwise comparisons with *P*-value < 0.1. We also highlighted comparisons with *P*-value < 0.05 by formatting them in bold for added emphasis. The *P*-values for multiple comparisons were reported only for statistically significant A/T/N group comparisons.

*(1)* A−/T−/N−; *(2)* A+/T−/N−; *(3)* A+/T+/N−; *(4)* A+/T+/N+; *(5)* A+/T−/N+; *(6)* A−/T+/N−; *(7)* A−/T+/N+; *(8)* A−/T−/N+.

*ANOVA* analysis of variance; *BMI* body mass index; *MMSE* Mini-Mental State Examination; *ADAS-cog J* Alzheimer-Disease Assessment Scale-Cognitive-Japanese version; *FAB* Frontal Assessment Battery; *WMS-R* Wechsler Memory Scale-revised.

**Supplementary Figure S1. Western blot analysis using an Apolipoprotein E isoelectric focusing system (JOKOH, Tokyo, Japan)**

**Supplementary Figure S2. ROC analysis by clinical diagnostic classification for each biomarker**

Lane ①：A−T−N−(E2/4)

Lane ②：A−T+N−(E3/3)

Lane ③：A−T+N−(E3/3)

Lane ④：A+T+N+ (E3/4)

　　　　④：20220214


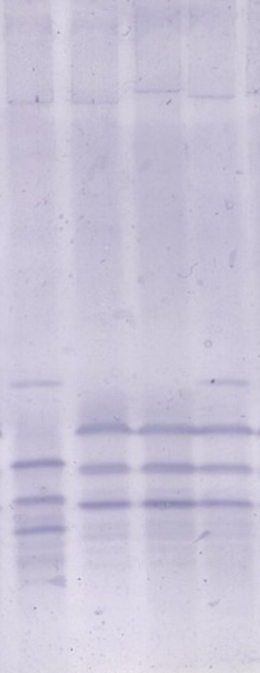


E4→

E3→

E2→

1. ② ③ ④


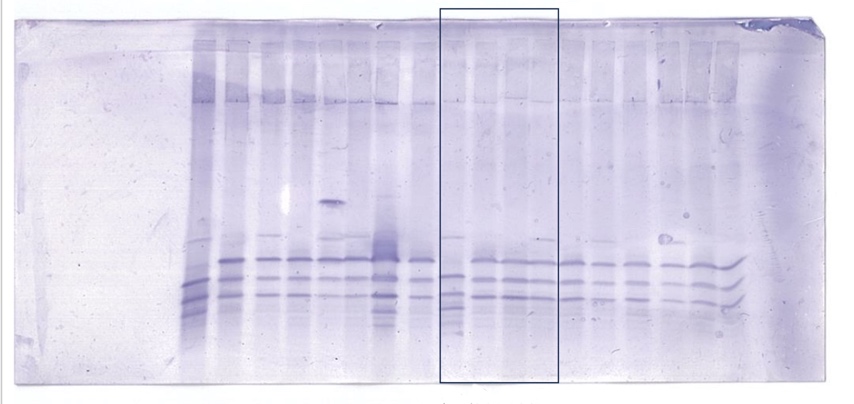


Whole gel

　　　　④：20220214

**Aβ42**

**p-Tau**

**t-Tau**

**Odor identification scores**

**Aβ42/40 ratio**

**p-Tau/Aβ42 ratio**

ROC analysis to discriminate (Normal + aMCI) vs AD.

AUC and cut-off values for AD diagnosis for each biomarker were established.

*Aβ* amyloid-β; *p-Tau* phosphorylated Tau; *t-Tau* total Tau; *ROC* receiver operating characteristic; *aMCI* amnestic mild cognitive impairment; *AD* Alzheimer’s disease; *AUC* area under the ROC curve.

**Supplementary Figure S3. One-way ANOVA by Kruskal Wallis test of neuropsychological testing and odor identification in each group of AD continuum of A/T/N and multiple comparison by pairwise Wilcoxon rank-sum test with Bonferroni correction**


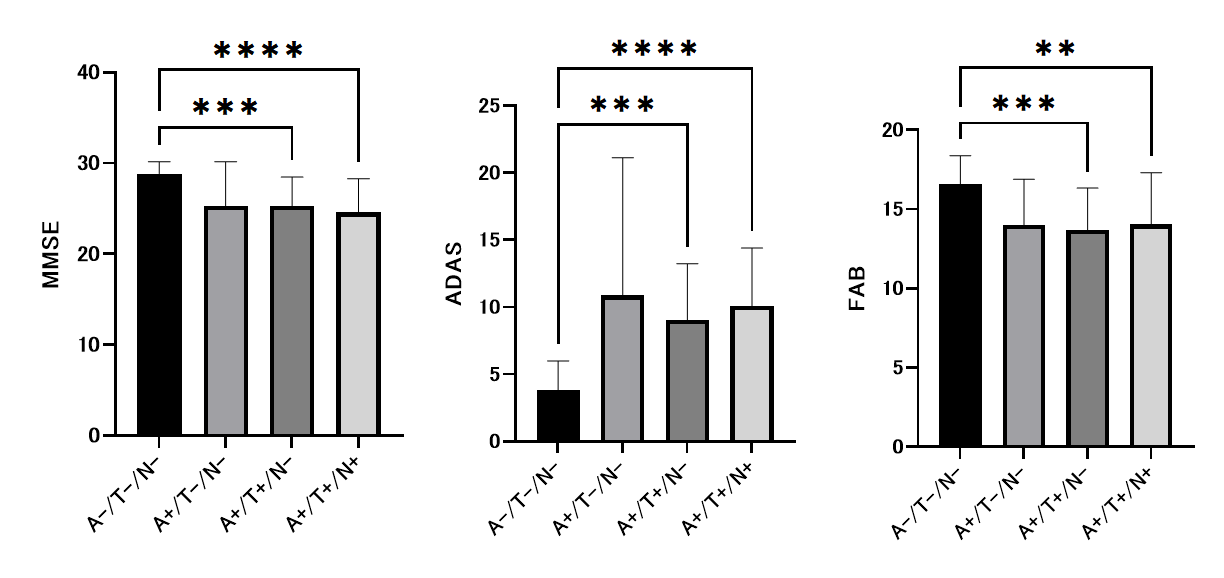

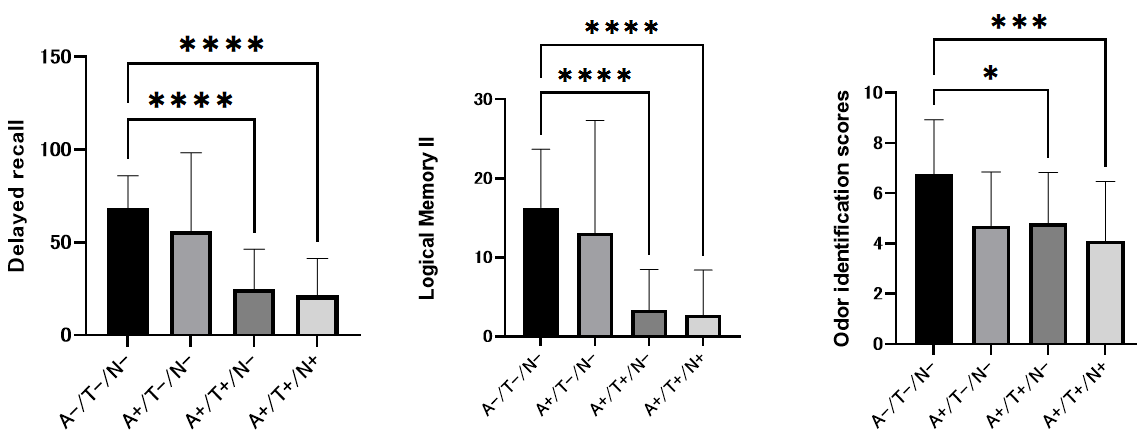


**MMSE (p<0.001) ADAS-cog J (p<0.0001) FAB (p=0.001)**

**Delayed recall Logical memory II Odor identification scores**

**(p<0.001) (p<0.0001) (p=0.001)**

**(p<0.0001) (p<0.0001) (p=0.003)**

Significant differences in MMSE, ADAS-cog J, FAB, Delayed recall, Logical memory-II, and odor identification scores occurred between A−/T−/N− and

A+/T+/N− and between A−/T−/N− and A+/T+/N+ groups, respectively. Odor identification scores, MMSE, ADAS-cog J, FAB, Delayed recall,

and Logical memory II worsened with neurodegeneration in the A/T/N classification.

*ANOVA* analysis of variance; *AD* Alzheimer’s disease; *MMSE* Mini-Mental State Examination;

*ADAS-cog J* Alzheimer-Disease Assessment Scale-Cognitive-Japanese version; *FAB* Frontal Assessment Battery.

**Supplementary Figure S4. Discriminability of A/T/N classification by the odor identification scores and ROC curves for setting** **of cutoff values**

AUC: 0.650

AUC: 0.670

No 5. SNAP

No 4. p-Tau accumulation

without amyloidogenesis

AUC: 0.717

AUC: 0.761

AUC: 0.716

No 3. AD neurodegeneration

No 1. amyloidgenesis

No 2. p-Tau accumulation


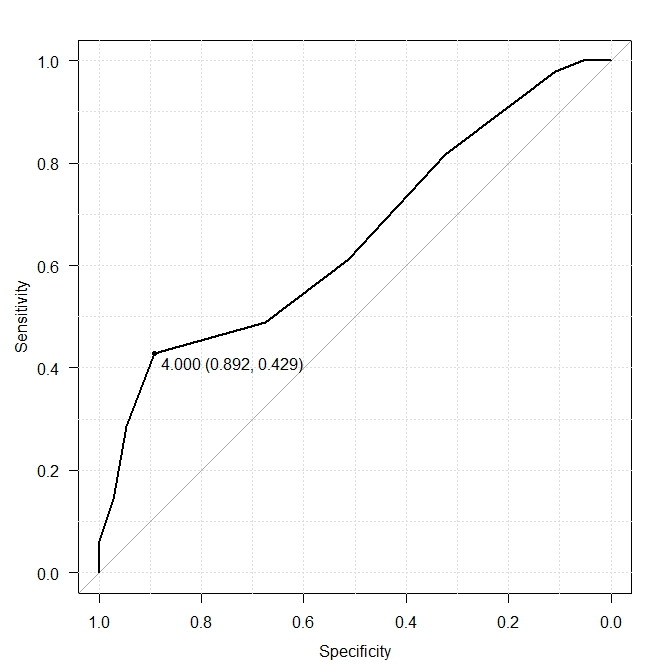


*ROC* receiver operating characteristic; *AUC* area under the ROC curve; *AD* Alzheimer’s disease.

**Supplementary Figure S5. ROC curves for examination of differences in AUCs of the odor identification scores and CSF biomarkers by the bootstrap method**

Odor identification score

CSF Aβ42/40

Odor identification score

CSF p-Tau 181/Aβ42

Odor identification score

CSF Aβ42

Odor identification scores vs. pTau181/Aβ42

Odor identification scores vs. Aβ42/40

Odor identification scores vs. Aβ42

Examination of differences in AUCs of the odor identification scores and CSF biomarkers by the bootstrap sampling method. Sampling frequency was 20,000. ROC analysis to discriminate (Normal + aMCI) vs. AD. To investigate whether olfaction can substitute for CSF biomarkers in AD diagnosis, we examined the difference in AUCs between the identification scores and each biomarker using the bootstrap method. Olfaction AUCs in AD were not significantly different from Aβ42, p-Tau, total Tau, Aβ42/Aβ40 ratio, or p-Tau/Aβ42 ratio. Therefore, odor identification scores did not differ significantly from these alternative markers. Therefore, odor identification scores are a potential surrogate for these markers.

*ROC* receiver operating characteristic; *AUC* area under the ROC curve; *CSF* cerebrospinal fluid; *AD* Alzheimer’s disease.

**Supplementary Figure S6. Comparison of AUC values for odor identification scores and CSF biomarkers between standard (12 items) and selected version (4 items) using ROC curves**


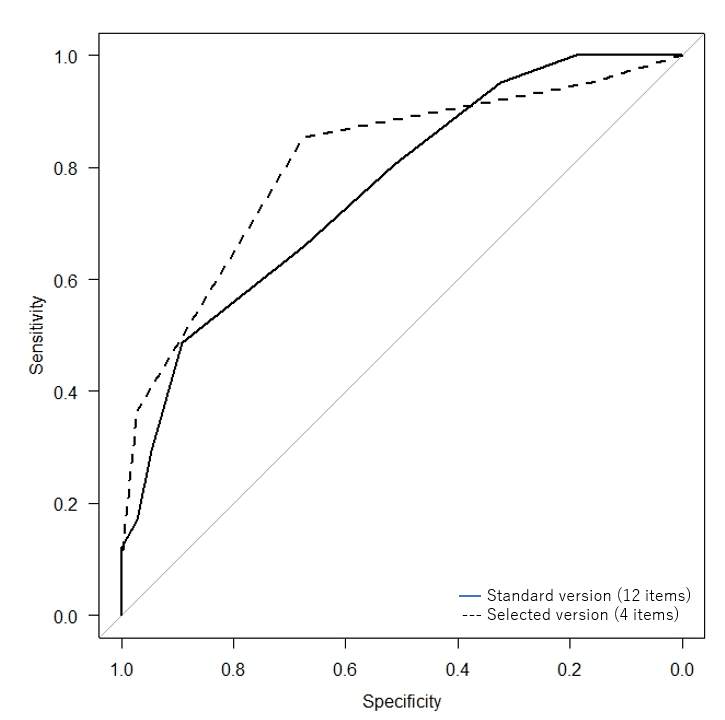


The selected version demonstrated similar specificity at 0.676 but showcased higher sensitivity (0.854 > 0.659) and a greater AUC value (0.809 > 0.761) compared to the standard version.

*AUC* area under the ROC curve; *CSF* cerebrospinal fluid; *ROC* receiver operating characteristic.
